# Marine-Derived Fucose-Containing Carbohydrates: Review of Sources, Structure, and Beneficial Effects on Gastrointestinal Health

**DOI:** 10.3390/foods13213460

**Published:** 2024-10-29

**Authors:** Xinmiao Ren, Shenyuan Cai, Yiling Zhong, Luying Tang, Mengshi Xiao, Shuang Li, Changliang Zhu, Dongyu Li, Haijin Mou, Xiaodan Fu

**Affiliations:** College of Food Science and Engineering, Ocean University of China, Qingdao 266404, China; renxinmiao1015@stu.ouc.edu.cn (X.R.); 18986507217@163.com (S.C.); zhongyiling321@163.com (Y.Z.); tangluying@stu.ouc.edu.cn (L.T.); 18227591863@163.com (M.X.); 15140923590@163.com (S.L.); chlzhu@163.com (C.Z.); lirong81@163.com (D.L.); luna_9303@163.com (X.F.)

**Keywords:** marine-derived fucose-containing carbohydrates, gastrointestinal health, gut microbiota, anti-pathogen, anti-inflammatory, structure–activity relationship

## Abstract

Fucose, fucose-containing oligosaccharides, and fucose-containing polysaccharides have been widely applied in the fields of food and medicine, including applications in *Helicobacter pylori* eradication and renal function protection. Fucose-containing carbohydrates (FCCs) derived from marine organisms such as seaweed, invertebrates, microalgae, fungi, and bacteria have garnered growing attention due to their diverse bioactivities and potential therapeutic applications. Marine-derived FCCs characterized by high fucose residue content and extensive sulfate substitution, including fucoidan, fucosylated chondroitin sulfate, and fucose-rich microbial exopolysaccharides, have demonstrated significant potential in promoting gastrointestinal health. This review describes the unique structural features of FCCs and summarizes their health benefits, including regulation of gut microbiota, modulation of microbial metabolism, anti-adhesion activities against *H. pylori* and gut pathogens, protection against inflammatory injuries, and anti-tumor activities. Additionally, this review discusses the structural characteristics that influence the functional properties and the limitations related to the activity research and preparation processes of FCCs, providing a balanced perspective on the application potential and challenges of FCCs with specific structures for the regulation of gastrointestinal health and diseases.

## 1. Introduction

l-fucose (6-deoxy-l-galactose) and d-fucose are a pair of fucose enantiomers that exist in nature [1]. As a rare monosaccharide, fucose has important physiological functions, including anti-cancer, anti-allergic, anti-coagulant, and anti-aging activities [2]. In recent years, fucose-containing carbohydrates (FCCs) have attracted increasing attention due to their abundant sources (plants, animals, and microorganisms) and functional activities [3]. Large quantities of fucosylated oligosaccharides, such as 2′-fucosyllactose, 3-fucosyllactose, difucosyllactose, and lacto-N-fucopentaose I, have been documented in human milk [4]. Fucosylated human milk oligosaccharides (HMOs) have been reported to play critical roles in regulating gut microbiota, improving gut barrier function, promoting immune development and tolerance, and enhancing brain development and cognition in infants [5,6,7].

Marine organisms, such as seaweed, echinoderms, microalgae, cyanobacteria, marine bacteria, and fungi, are rich sources of FCCs [8,9,10,11]. These biomolecules play a crucial role in the survival of these organisms in the harsh marine environment by providing protection against extreme conditions like low temperatures, high salinity, and elevated pressure [12,13]. The presence of fucosylated side chains confers resistance to degradation by enzymes, such as chondroitinases, secreted by marine microorganisms [14]. The high sulfate content of most marine-derived FCCs is essential for their survival in the marine environment, as it increases their charge density and enhances their stability [15]. Sulfated fucans, including fucoidan and galactofucan sulfate (GFS), are composed of sulfated l-/d-fucose and varied monosaccharides, such as galactose, mannose, glucose, xylose, and uronic acids, and their structural diversity is influenced by factors like species, climate, geographic location, and extraction methods [16,17,18,19,20]. Fucosylated chondroitin sulfate (FCS) shares a similar backbone structure with mammalian chondroitin sulfate, consisting of alternating α-glucuronic acid (GlcA) and *N*-acetyl galactosamine residues (GalNAc), but with the addition of l-fucosyl units linked to the acid residues [21]. While the ratio of GalNAc to GlcA remains relatively constant across different FCS-producing species, the proportions of sulfates and fucose can vary significantly [8,14]. FCCs from various marine bacteria, fungi, microalgae, and cyanobacteria are often found in l-/d-fucose-rich exopolysaccharides (EPS) produced and secreted by these microorganisms in response to environmental stress [9].

The gastrointestinal (GI) system is essential for digestion, absorption, metabolism, and overall human health. The global burden of GI diseases (GDs) is escalating, with gastric and colorectal cancers ranking among the top cancer-related deaths [22]. The prevalence of GDs, including peptic ulcer, inflammatory bowel disease (IBD), ulcerative colitis, and GI cancer, is rising [23,24]. Furthermore, non-GI diseases like obesity and cardiovascular disease are linked to intestinal microflora (microbiota) and GI health [25,26]. Consequently, interest in GI health is growing. FCCs exhibit diverse biological activities, including antiviral, antioxidant, anticoagulant, anti-inflammatory, and anticancer properties, leading to a wide range of applications in functional foods, pharmaceuticals, and cosmetics [27,28,29,30,31]. Various oral formulations of fucoidan and FCS are widely used as therapeutic drugs or dietary supplements in China, Australia, Russia, Republic of Korea, and Japan [32,33,34]. As non-digestible carbohydrates, FCCs reach the distal colon, where they influence the gut microbiota composition [8,32]. Emerging evidence demonstrated FCCs’ comprehensive effects on GI health, including shaping gut microbial communities, enhancing barrier function, and exerting anti-inflammatory and anti-tumor activities within the GI system [35,36,37,38].

In this review, the diversity of the structural and chemical compositions of marine-derived FCCs is categorized and described by source species. It delves into the beneficial impacts of FCCs on gut microbiota, microbial metabolism, and various health regulatory effects on gastrointestinal diseases, drawing upon existing research in the field. Furthermore, this review briefly discusses the structural characteristics that influence their functional properties and the challenges related to their activity research and preparation processes, providing a valuable framework for novel structures development and high-value functional applications of marine FCCs.

## 2. Sources and Chemical Structure of Marine-Derived Fucose-Containing Carbohydrates

**Figure 1:**
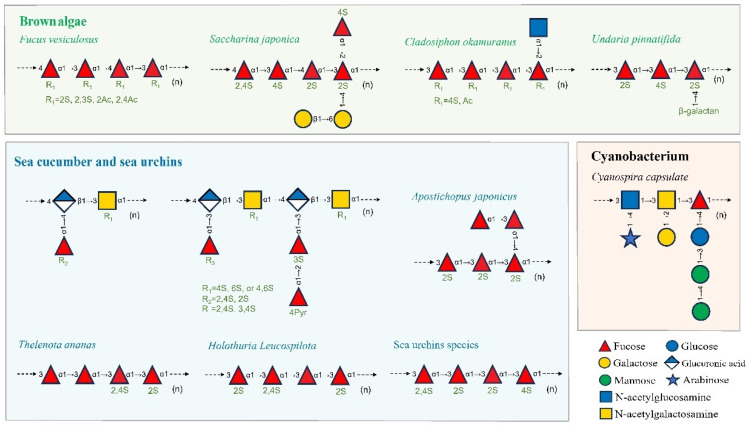
The structures of marine-derived fucose-containing carbohydrates including sulfated fucans, fucosylated chondroitin sulfate, and fucose-rich EPS from certain species of brown algae (light green box bottom), sea cucumber and sea urchins (light blue box bottom), echinoderms, and cyanobacteria (light red box bottom). Graphical symbols are depicted according to the symbol nomenclature for glycans (SNFG).

The primary marine sources of FCCs include seaweed, echinoderms, and certain species of microalgae, cyanobacteria, bacteria, and fungi, with the main types consisting of sulfated fucans (fucoidan, GFS, etc.), FCS, and fucose-rich EPS [13,39,40]. FCCs from different sources exhibit diverse compositions and chemical characteristics, including monosaccharide composition, glycosidic linkages, sulfate substitutions, and acetyl group modifications. Figure 1 illustrates the types and structural features of FCCs in various species. These insights are considered beneficial for understanding the structure–activity relationships of FCCs and developing targeted functional products.

FCCs derived from seaweed primarily consist of cell wall polysaccharides and their oligosaccharide derivatives, such as fucoidans from brown algae. Fucoidans are characterized by a backbone composed of α(1→3)-linked l-fucopyranose residues or alternating α(1→3) and α(1→4)-linked l-fucopyranosyls, often with sulfate, acetate, and acetyl group substitutions [8,41,42]. Beyond fucose, the monosaccharide composition of fucoidan extracts from various seaweed species includes hexoses (glucose, xylose, galactose, and mannose) and uronic acids (GlcA and galacturonic acid). Fucoidan preparations from edible brown algae species, such as *Fucus vesiculosus*, *Apostichopus japonicus* (synonym *Saccharina japonica*), *Cladosiphon okamuranus*, and *Undaria pinnatifida*, are commercially available, with production levels reaching up to 250 mg per gram dry weight [41]. Fucoidan from *F. vesiculosus* typically consists of a backbone of alternating α(1→3) and α(1→4)-linked l-fucopyranosyls with sulfate groups on O-2 and/or O-3 [16]. Fucoidan extracted from *A. japonicus* is primarily a GFS, containing fucose and galactose with sulfate substitutions and small amounts of other monosaccharides. The GFS backbone always comprises α(1→3)- or α(1→4)-linked α-l-fucopyranose with β-d-galactose branches at the C-4 position of the 3-linked α-l-fucopyranose residues and sulfate substitutions on the O-2 and/or O-4 positions [43]. Compared to *A. japonicus*, GFS from *U. pinnatifida* exhibits a higher molar ratio of galactose to fucose (1.0:1.1) [44]. The main structural units of GFS from *U. pinnatifida* include (1→3)(1→4)-linked α-Fuc*p* and (1→3)(1→4)(1→6)-linked β-Galp with sulfate substitutions on the O-2 or O-4 positions of fucose residues and the O-3 or O-6 positions of galactose residues [10,39,44,45]. Fucoidans from *C. okamuranus* are characterized by acetylated substitutions, containing linear backbones of α(1→3)-linked l-fucopyranose with some fucose residues being 2-O-acetylated and sulfate substitutions on the O-4 position [46]. The GlcA or fucose residues branching from the main chain are attached to the C-2 position of the non-sulfated fucose residues [18,46].

The molar ratio of fucose in the cell wall polysaccharides of red and green algae is approximately 1%, suggesting that fucose is not a core structural component of red algal sulfate galactans or green algal ulvan [47,48]. While fucose is not a major monosaccharide component of red and green algae carbohydrates, several studies have reported the presence of fucose-containing carbohydrates in these algae [49]. For instance, Lim et al. (2009) identified an antioxidant substance rich in fucose (20 mol%) from the red alga *Gloiopeltis tenax,* featuring a branched linkage of α-d-Man(1→3) α-d-Fuc, where the fucose residues were 2-O- and 4-O-sulfated [49]. Additionally, the polysaccharides isolated by Zhang et al. (2013) and Barakat et al. (2022) from the green algae *Enteromorpha linza* and *Ulva fasciata* were notably rich in fucose, constituting the second highest molar ratio in their monosaccharide composition, exceeding 20% [50,51].

Invertebrates, such as sea cucumbers and sea urchins, contain two primary types of active carbohydrates with fucose, including sulfated fucans and FCS. Sulfated fucans have been reported as the components of extracellular matrix proteoglycans found in the body walls of sea cucumbers and the egg jelly of sea urchins [52]. Sulfated fucans from invertebrate are typically linear, fairly regular, and repetitive in structure, compared to seaweed-derived fucoidan [15]. Sulfated fucans and their derived oligosaccharides from sea cucumbers often consist of mono-, tri-, or tetrasaccharide repetitive blocks of α(1→3)-linked or α(1→4)-linked l-fucopyranose residues, with sulfate substitutions at the *O*-2 and/or *O*-4 positions of the fucose residues [53]. While most fucoidans from sea cucumber species (e.g., *Thelenota ananas* and *Holothuria leucospilota*) are linear polysaccharides, some species, like *A. japonicus*, exhibit branched structures with long fucose units such as [α-l-Fuc*p*2(OSO_3_^−^)-1→3, (α-l-Fuc*p*-1→4-α-l-Fuc*p*-1→)4-α-l-Fuc*p*2(OSO_3_^−^)-1→3-α-l-Fuc*p*2(OSO_3_^−^)]_n_ [54]. The structure of sulfated fucans from sea urchins is similar to that of sea cumber, with a higher sulfate substitution rate on fucose residues, according to the studies on elucidated structures of sulfated fucans from several species of sea cucumbers and sea urchins [11].

FCS, primarily composed of GlcA, GalNAc, fucose, and sulfate, is abundant in invertebrates, particularly sea cucumbers, and exhibits various pharmacological properties. Compared to mammalian chondroitin sulfate, marine invertebrate chondroitin sulfate extracts often contain multiple fucose residues, with a slightly higher fucose content than GlcA or GalNAc [55]. In the basic FCS model, Fuc_2S4S_, Fuc_3S4S_, and Fuc_4S_ side chains are attached to the *O*-3 position of GlcA in the main chain [40]. FCS from *Isostichopus badionotus* consists of repeating units of →3)-d-GalNAc-(β1,4)-d-GlcA_4S6S_-(β1→ with a branch of (α1,2)-l-Fuc_2S4S_ connected to the *O*-3 position of the GalNAc residue [56]. Recent studies suggest that fucose side chains can also be attached to the *O*-2, *O*-4, or *O*-6 positions of GalNAc [21].

EPS is produced and secreted by many marine microorganisms, such as bacteria, fungi, microalgae, and cyanobacteria, for resisting environmental stress [57]. Fucose, a component of EPS secreted by microorganisms in extreme environments such as high/low temperatures, high salinity, high pressure, and oligotrophic conditions, plays a crucial role in cryoprotection, water-holding capacity, and thermostability [12,13,46]. A thermostable EPS, EPS1-T14, produced by *Bacillus licheniformis* T14, an alkalophilic and thermophilic bacterium isolated from a submarine vent in Panarea Island (Italy), primarily consists of fructose, fucose, and glucose in a molar ratio of 1.0:0.75:0.28 [12]. The fucose in EPS1-T14 contributes significantly to its thermostability, maintaining stability up to 80 °C. The EPS of *Polaribacter* sp. SM1127, isolated from the Arctic brown alga *Laminaria*, contains terminally linked fucopyranose with a fucose content of 7.4 mol%, exhibiting superior moisture-retention properties compared to hyaluronic acid, chitosan, sodium alginate, and glycerol [13]. According to a previous study of EPS from marine microalgae and cyanobacteria, fucose is the primary or secondary monosaccharide in EPS from the Charophya and Ochrophyta phyla, while species of cyanobacteria and other phyla are not fucose-rich EPS producers [9]. Compared to large algae and invertebrates, fucose-rich EPSs are generally neutral heteropolysaccharides with repeating units of more than three monosaccharide types, low sulfate substitution levels, and complex branched structures [3]. The structure of EPS from the cyanobacterium *Cyanospira capsulate* has been characterized by Garozzo et al. (1998), revealing a complex repeating unit of [3)-, (α-l-Ara*p*-1→)4-α-d-Glc*p*NAc-1→3, (α-l-Gal*p*A-1→)2-α-l-Gal*p*A-1→3, ((S)-CH_3_CO_2_HCH-4-β-d-Man*p*-1→4-β-d-Man*p*-1→3-β-d-Glc*p*-1→)-α-l-Fuc*p*-(1→]_n_ [58].

## 3. The Impact of Marine-Derived Fucose-Containing Carbohydrates on the Gastrointestinal Health

**Figure 2:**
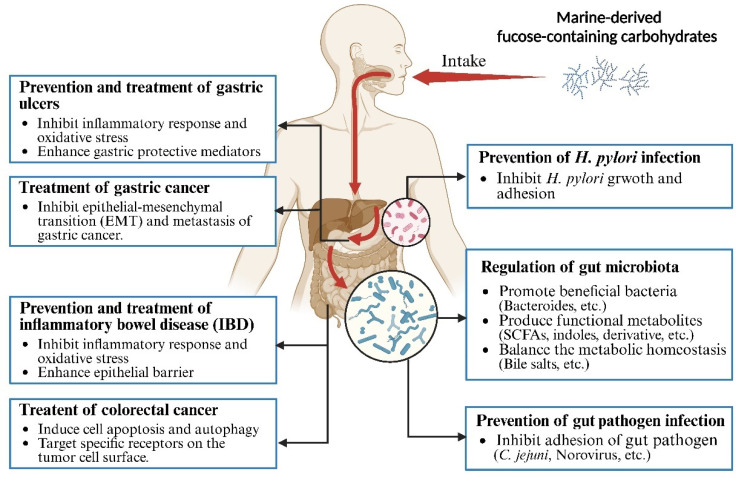
The beneficial effects of marine-derived fucose-containing carbohydrates on regulation of gut microbiota, anti-adhesion against pathogens, and prevention/treatment of gastrointestinal diseases.

There has been a global surge in the number of individuals at risk for gastrointestinal diseases [59]. Risk factors for these diseases include genetic predisposition, obesity, alcohol consumption, poor diet, chronic stress, aging, metabolic disorders, non-steroidal anti-inflammatory drug use, and pathogen infections [59,60]. Gastrointestinal diseases, such as *Helicobacter pylori* infection, peptic ulcers, infectious diarrhea, IBD, and gastrointestinal cancers, affect individuals of all ages [61,62,63]. Current treatment strategies for gastrointestinal diseases, including antibiotics, immunosuppressants, and chemotherapeutic drugs, exhibit limitations due to the development of resistance and adverse side effects [64]. The global rise in antibiotic resistance has significantly reduced the effectiveness of *H. pylori* treatment, potentially increasing the risk of complications like peptic ulcers and gastric cancer [65]. In recent years, natural polysaccharides have emerged as promising candidates for treating and preventing diseases due to their high activity, low toxicity, and minimal side effects [66,67,68]. As a non-digestible carbohydrate, FCCs selectively act as a substrate for host microbiota, conferring health benefits [69]. Moreover, FCCs exhibit anti-inflammatory, antioxidant, and anti-tumor activities through pharmacological mechanisms involving target molecules and downstream signaling pathways [70]. Figure 2 and Table 1 summarize the effects of FCCs on gastrointestinal microbiota and diseases, including the regulation of gut microbiota, anti-pathogen adhesion, prevention and protection against gastrointestinal damage, and anti-gastrointestinal cancer activities.

### 3.1. Anti-H. pylori Activities

As a common bacterial pathogen in the stomach, *H. pylori* is known for its ability to survive in acidic gastric juices and colonize the gastric mucosa. *H. pylori* is a primary risk factor for chronic gastritis, peptic ulcer disease, mucosal tissue-associated lymphoma, and gastric cancer, and was classified as a class I carcinogen by the World Health Organization in 2017 [99]. Based on growing research into the pathogenic targets of *H. pylori*, FCCs have been considered as potential drug candidates for the eradication therapy of *H. pylori* infection.

**Figure 3:**
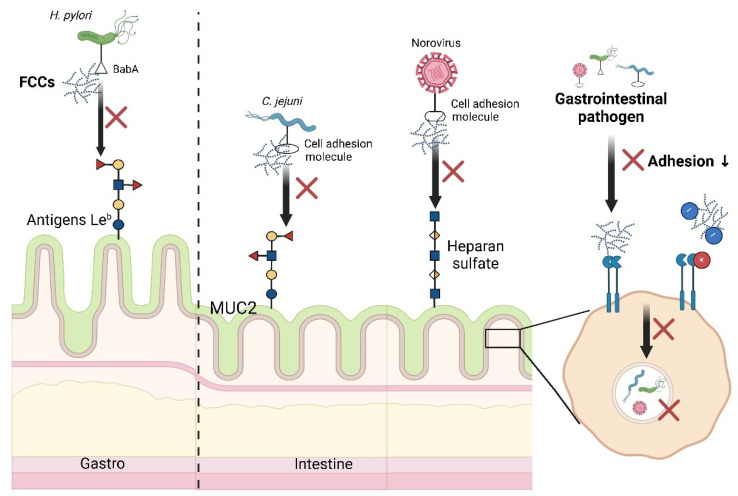
Anti-adhesive strategies of marine-derived fucose-containing carbohydrates against gastrointestinal pathogens. FCCs have been shown to have anti-pathogenic potential by binding to pathogens and/or epithelial receptors as anti-adhesive molecules. MUC2, mucin 2.

Preventing interactions between *H. pylori* ligands and host cells receptors is one of the critical targets in treating *H. pylori* infections (Figure 3). Fucoidan inhibits *H. pylori* adhesion to host cells in the gastric adenocarcinoma AGS cell and BALB/c mouse models. Mechanistically, fucoidan binds to *H. pylori* adhesins BabA and AlpA, reducing infection rates by 60%. The outer membrane protein BabA binds to fucosylated histo-blood group antigens Le^b^ found on gastric epithelial cells and mucin [100]. The Le^b^ oligosaccharide structure contains two fucose residues, two galactose residues, an GlcNAc, and a glucose residue, partially overlapping with the monosaccharide composition and glycosidic linkages of FCCs (Figure 1) [101]. It is now understood that the sulfate groups in fucoidan effectively inhibit *H. pylori* attachment to porcine gastric mucin under acidic conditions (pH = 2) [76]. Compared to non-sulfated polysaccharides like dextran, sulfated polysaccharides, including fucoidan (from *Cladosiphon* and *Fucus*) and dextran sulfate sodium, exhibited significant anti-adhesive effects under acidic conditions, demonstrating their potential for infection prevention of *H. pylori*. Pre-treatment with fucoidan significantly reduced *H. pylori* infection rates in Mongolian gerbils compared to post-infection treatment. 

Clinical studies have evaluated the efficacy of a compounded fucoidan drink (FPD) for *H. pylori* eradication. After 8 weeks of FPD administration, the *H. pylori* eradication rate was 80.5%, and the clearance rate (negative conversion) was 26.8%, only 12% lower than quadruple therapy [77]. Gastric mucosa inflammation is a common result of the interaction between *H. pylori* and gastric epithelial cells [102]. Fucoidans from various algal species have demonstrated anti-ulcer effects in peptic ulcer rats infected with *H. pylori*, with recovery rates ranging from 21.1% to 49.1% [103]. The number of gerbils with gastric mucosal edema decreased in response to fucoidan treatment compared with the *H. pylori* infected untreated group [76].

### 3.2. Regulatory Effects on Gut Microbiota Composition

**Figure 4:**
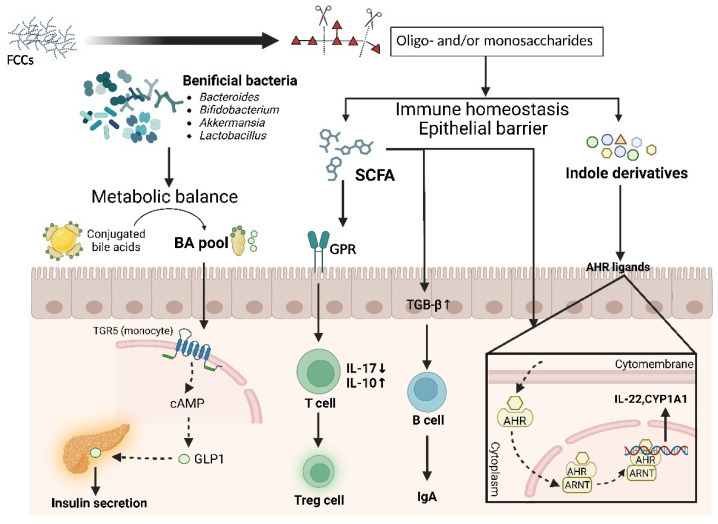
Gut microbiota metabolism of marine-derived fucose-containing carbohydrates and signal transduction pathways related to SCFAs, indole derivatives, and bile acids. FCCs increase the numbers of beneficial bacteria and levels of beneficial metabolites, which confers a favorable balance in the gut microbiota and systemic metabolism. BA, bile acids; cAMP, cyclic adenosine monophosphate; GLP1, glucagon-like peptide-1; GPR, G protein-coupled receptors; TGB-β, transforming growth factor-β; IgA, immunoglobulin A; AHR, aryl hydrocarbon receptor; ARNT, aryl hydrocarbon receptor nuclear translocator; CYP1A1, cytochrome P450 1A1.

Maintaining the homeostasis of the gastrointestinal microbiota is essential for host health, while gut dysbiosis can increase the risk of various diseases, including inflammatory bowel disease, diabetes mellitus, cardiovascular diseases, and cancers [104,105,106]. Numerous studies support the beneficial effects of FCCs on the gut microbiota in animal models and human clinical trials [72,73,74,107]. As a non-digestible carbohydrate, FCCs are fermented by resident microbiota in the distal colon after oral ingestion, increasing the abundance of beneficial bacteria (e.g., *Ruminococcus*, *Bacteroides*, *Bifidobacteria*, *Akkermansia*, *Lactobacillus*, and *Desulfovibrio*) and beneficial metabolites, such as short-chain fatty acids (SCFAs), indole derivatives, and secondary bile acids, which contribute to maintaining a favorable balance in the gut microbiota and systemic metabolism (Figure 4).

Fucoidan and FCS have been shown to promote the growth of beneficial bacteria, such as *Bacteroides* and *Bifidobacteria*, in in vitro fermentation models using human fecal samples. Fu et al. (2024) found that fucoidan extracted from *Laminaria japonica* and its derived oligosaccharides increased the abundance of *Bacteroides*, *Bifidobacterium*, *Lactobacillus*, and *Megamonas* [71]. Compared to fucoidan, fucoidan-derived oligosaccharides exhibited a stronger promotive effect on *Bifidobacterium* and *Lactobacillus* [71]. FCS has been reported to modulate the gut microbiota community structure by increasing the abundance of beneficial bacteria (e.g., *Megamonas*, *Bacteroides*, *Fusobacterium*, *Parabacteroides*, *Prevotella*, and *Faecalibacterium*) and reducing pathogenic bacteria [8]. 

Growing evidence suggests that sulfated fucans can prevent intestinal microbiota disorders in various metabolic diseases. While the actual regulatory effects of carbohydrates on microbiota structure are influenced by their structure and the specific microbiota composition of the disease model, it is well-documented that sulfated fucan interventions can prevent gut microbiota dysbiosis induced by diseases [19,42,72]. Fucoidan significantly improved gut microbiota dysbiosis during standard quadruple therapy for eradicating *H. pylori*, including the restoration of normal *Bifidobacterium* abundance [73]. Administration of fucoidans from *L. japonica* to high-fat diet (HFD)-fed mice increased the levels of *Bacteroides, Akkermansia*, and *Desulfovibrio*, accompanied by improvements in metabolic and inflammatory alterations induced by the HFD [42]. Higher Firmicutes/Bacteroidetes (F/B) ratios are generally observed in obese individuals, while an increase in Bacteroidetes can significantly improve this disease indicator. In another study using HFD rats supplemented with fucoidan from *U. pinnatifida*, the abundance of *Bacillus*, *Ruminococcus*, *Adlercreutzia*, *Prevotella*, *Oscillospira*, *Enterobacter*, and *Desulfovibrio* increased in the fucoidan group compared to the non-supplemented HFD group [72]. A similar study on HFD rats supplemented with fucoidan from *Sargassum fusiforme*, which consists of α-l-Fuc and β-d-Galp, showed a significant increase in the abundance of *Bifidobacterium* in addition to *Bacteroides* [19]. FCS have also been reported to improve gut microbiota dysbiosis in HFD-fed mice by reducing the F/B ratio. Li et al. (2019) and Hu et al. (2019) found that FCS from *Isostichopus badionotus* and *Acaudina molpadioides* improved gut microbiota dysbiosis in HFD-fed mice by decreasing the abundance of Firmicutes and increasing the abundance of Bacteroidetes, especially *Bacteroides* [30,56]. FCS from *Holothuria leucospilota* increased the abundance of SCFAs-producing bacteria, leading to improved type 2 diabetes mellitus conditions [74].

### 3.3. Modulation Effect on Metabolism of Gut Microbiota 

FCCs have been reported to be metabolized by beneficial bacterial genera, such as *Bacteroides*, into oligosaccharides and monosaccharides, leading to the production of SCFAs [42,74]. SCFAs, which serve as signaling molecules, are closely linked to the regulation of intestinal inflammation and the maintenance of gut immune homeostasis. SCFA-induced inhibition of histone deacetylases in T cells enhances the acetylation of p70 S6 kinase and phosphorylation of rS6, modulating the mTOR pathway necessary for IL-10 production and alleviating inflammation [108,109]. Additionally, SCFAs (butyrate and acetate) enhance gut immunity by increasing the expression of TGF-β in intestinal epithelial cells or aldehyde dehydrogenase 1 family member A2 in dendritic cells [110]. In the metabolomics of fecal fermentation of fucoidan, indole derivatives such as indole-3-acetic acid were identified as metabolic markers of the fucoidan group [71]. Laursen et al. (2021) found a significant positive correlation between the high abundance of *Bifidobacterium* and the production of aromatic amino acid metabolites such as indole derivatives [111]. Indole and its derivatives are recognized as ligands of the aryl hydrocarbon receptor, which participates in epithelial barrier protection and immune homeostasis [112]. Moreover, FCCs show a potential effect in restoring gut metabolic balance disrupted by disease (Figure 4). Fucoidan improved the decreased bacterial bile salt hydrolase activities induced by the HFD in the ileum. Unconjugated bile acids (BAs) and secondary BAs, catalyzed by a series of enzymes in the gut microbiota, are critical signaling molecules for the homeostasis of the BA pool. The binding of secondary BAs with BA receptors such as FXR and TGR5 maintains lipid (cholesterol and triglyceride) homeostasis and glucose homeostasis [72].

### 3.4. Anti-Infection Strategies Against Pathogenic Bacteria

A common feature of gastrointestinal pathogens is their ability to penetrate and disrupt the mucosal barrier, reaching the epithelial cells and compromising tight junctions. This disruption allows pathogens to enter underlying tissues and cause acute or chronic inflammation [113]. Many pathogens are chemoattracted to mucus and use it as a signal to upregulate virulence factors, including glycosidases, proteases, toxins, and invasive antigens, facilitating evasion of the mucus barrier [114,115]. Special types of carbohydrates, such as chitosan and fucosylated HMOs, have been reported to be important inhibitors of pathogenic infection development, exerting anti-pathogenic effects [116].

Marine fucoidan compounds, characterized by fucose residues and sulfate groups, have been shown to inhibit the attachment of pathogenic particles to host cell surfaces. The fucose residue backbones act as decoy receptors for pathogens, mimicking the structure of host cell receptor molecules (Figure 3). *Campylobacter jejuni,* a representative intestinal pathogen, commonly causes bacterial diarrhea and can lead to serious complications such as Guillain–Barré syndrome, reactive and septic arthritis, and Miller–Fisher syndrome, particularly in individuals with compromised immune systems, underlying diseases, or serious infections [117]. The preferred niche of *C. jejuni* is the crypts of the intestinal epithelium, which are rich in the gel-forming mucin MUC2, a protein characterized by α(1→3)-linked/α(1→4)-linked α-l-fucose branches and sulfate substitution at the *O*-2 or *O*-4 positions of galactose/GalNAc [118,119]. Recent research has demonstrated that fucoidan oligosaccharides are glycomimetic ligands specifically recognized by *C. jejuni*, effectively blocking bacterial adhesin–carbohydrate recognition and preventing the binding of *C. jejuni* to intestinal epithelial cells [78]. A neoglycan, synthesized from fucoidan oligosaccharides and bovine serum albumin, can be selectively recognized by both *Ulex europaeus* fucose-specific lectin and viable *C. jejuni*, while no interactions were observed between *Escherichia coli* and this fucosylated glycoconjugate. Additionally, negatively charged biopolymers directly interact with the surface of these pathogens. For host cells, the polyanions bind to positively charged host cell receptors, forming an anti-adhesion barrier. Compared to components with low sulfate and uronic acid content, fucoidan with lower molecular weight (Mw) and stronger polyanionic characteristics enhanced activity against *E. coli* [120]. The highly electronegative fucoidan components not only bind to bacterial membrane proteins but also alter their membrane structure.

### 3.5. Antiviral and Antifungal Properties

Sulfated polysaccharides and oligosaccharides have been reported to exhibit antiviral activity against a wide range of viruses [27,81,121]. In vitro and in vivo studies with fucoidan have demonstrated broad-spectrum antiviral effects, effectively inhibiting infections from viruses such as hepatitis, influenza, human immunodeficiency virus (HIV), severe acute respiratory syndrome coronavirus 2 (SARS-CoV-2), bovine viral diarrhea, herpes simplex virus, and human papillomavirus [122,123]. Furthermore, low Mw fractions of fucoidan have also shown antiviral activity, effectively inhibiting Type I influenza virus, adenovirus, and parainfluenza virus in vitro [124]. Fucoidan has been specifically highlighted for its inhibitory effects on norovirus, the primary cause of non-bacterial epidemic gastroenteritis worldwide, accounting for over 95% of gastroenteritis cases. In vitro experiments demonstrated that GFS prevented the binding of norovirus GII.4 virus-like particles to histo-blood group antigens by altering the morphological structure of the viral particles [81]. Fucoidan has also been reported to enhance the innate immunity of zebrafish larvae against human norovirus [80]. FCS showed diverse antiviral activities against viruses, including HIV, SARS-CoV, and beta-herpesvirus, through the competitive inhibition of virion binding to heparan sulfate [27,125]. Heparan sulfate is a sulfated glycosaminoglycan present on the cell surface that plays a crucial role as a co-receptor in many viral infections [126]. However, the main drawback in developing FCS into novel clinical antiviral candidates is its anticoagulant properties. Marine-derived FCCs have shown antifungal properties against several genera, such as *Candida*, *Aspergillus*, and *Mucor* owing to the structural foundation of sulfated substitution and a sugar backbone. Fucoidan extracted from *Cystoseira barbata* inhibited the growth of *C. albicans*, *C. glabrata*, and *C. parapsilosis* with minimum fungicidal concentrations as low as 0.1 μg/mL [127]. Fucoidan from *U. pinnatifida* has been reported to exhibit antifungal activity against *A. flavus*, *A. fumigatus*, and *Mucor* spp. [31].

### 3.6. Protection of Inflammatory Injuries

**Figure 5:**
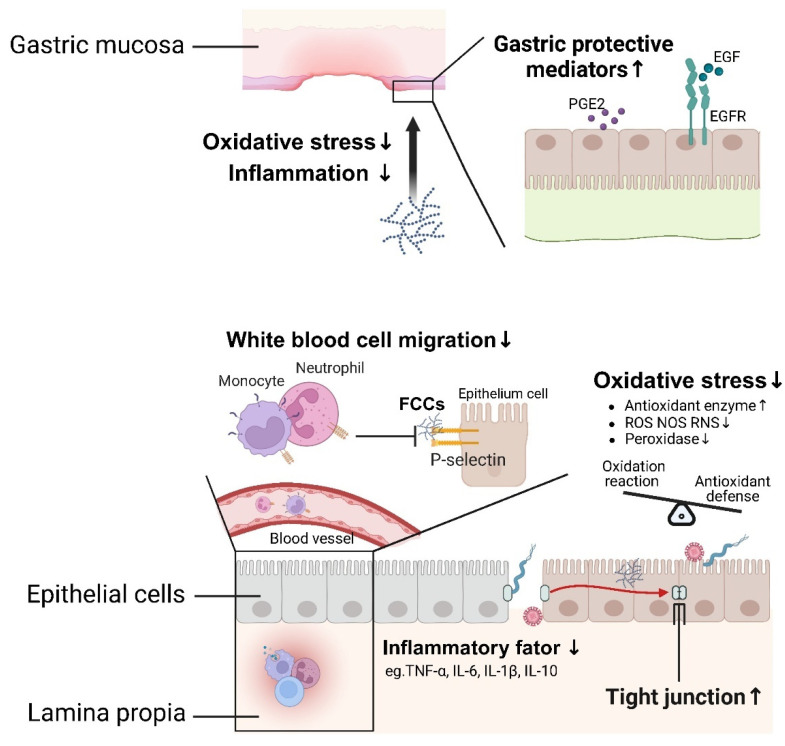
Prevention and protection of marine-derived fucose-containing carbohydrates on inflammatory injuries. FCCs ameliorate peptic ulcers and IBD through modulation of cytokines, protective mediators, leukocyte migration, balance between oxidative reactions and antioxidant defenses, and tight junctions. PGE2, prostaglandin E2; EGF, epidermal growth factor; EGFR, epidermal growth factor receptor; ROS, reactive oxygen species; RNS, reactive nitrogen species.

Risk factors, such as drug exposure, pathogen infections, psychological stress, or chemicals, overwhelm protective factors/mechanisms and the mucosal barrier may become injured, leading to immune system disturbance and imbalanced interactions with microbes. This can result in the development of acute or chronic inflammation in the gastrointestinal tract of the host [128]. Prevention and protection of gastrointestinal injuries by FCCs are illustrated in Figure 5. Mucosal damage, a common type of gastrointestinal injury, is clinically recognized as an acute or chronic peptic ulcer. This condition is characterized by a rupture of the mucosal barrier that extends into the muscle layer, forming a cavity surrounded by inflammation.

Mucosal lesions represent the most common type of gastrointestinal damage and are clinically referred to as peptic ulcers. These ulcers are characterized by breaches in the mucosal barrier that extend into the muscular layer, forming cavities surrounded by acute or chronic inflammation [129]. Studies in both rat models and clinical settings have demonstrated the preventive and therapeutic effects of fucoidan against gastric ulcers induced by *H. pylori*, non-steroidal anti-inflammatory medications, and alcohol [75,85,130]. Fucoidan attenuates ethanol-induced gastric mucosal damage in rats through mechanisms involving antioxidant, anti-inflammatory, and pro-survival properties. Research by Selim et al. (2023) and Choi et al. (2010) indicated that the anti-ulcer properties of fucoidan might depend on the positive regulation of numerous inflammatory cytokines, including tumor necrosis factor (TNF)-α, interleukin (IL)-6, IL-1β, and IL-10, along with pro-inflammatory apoptosis mediators such as caspase-1 and gasdermin D [84,85]. Pretreatment with fucoidan counteracted ethanol-induced oxidative stress by reducing malondialdehyde levels and increasing gastric superoxide dismutase (SOD) activity and glutathione levels. Additionally, levels of gastric protective mediators, such as prostaglandin E2 (PGE2), epidermal growth factor (EGF), and EGF receptor, which are known to promote ulcer healing, were also elevated through alginate intervention [86]. In clinical trials, fucoidan from *C. okamuranus* has been reported to promote ulcer healing and alleviate symptoms such as vomiting and abdominal pain by inducing epithelial cells to produce growth factors [87,130].

The disturbance of the gut immune system and microbiota contributes to the development of IBD in susceptible individuals [131]. Conventional treatments, including anti-inflammatory drugs, immunosuppressants, and surgical procedures, show serious side effects and lead to drug resistance. Consequently, multi-target natural bioactive compounds, such as marine algae polysaccharides, are being explored as new treatment strategies to improve the clinical symptoms of IBD. It has been well-established that the cytokine spectrum is a potential target for the treatment of intestinal inflammation [132]. Various pro-inflammatory mechanisms and mediators have been reported to be regulated by fucoidans [85,88,133,134,135]. Tauseef examined the protective effects of oral administration of fucoidan on the structural integrity and architecture of the colon [135]. Various fucoidan extracts obtained from different seaweed sources have been shown to reduce the production of pro-inflammatory cytokines, including TNF-α, IL-1β, IL-6, IL-10, interferon-gamma (IFN-γ), macrophage inflammatory protein-1 alpha (MIP-1α), MIP-1β, granulocyte colony-stimulating factor (G-CSF), and GM-CSF, thereby inhibiting colonic inflammation [128,136]. Epithelial dysfunction and patient symptoms in inflammatory intestinal diseases have been reported to be associated with the migration and involvement of leukocytes in places of inflammation, which is mediated by adhesion molecules such as selectins, integrins, and cadherins [136]. Fucoidans, identified as high-affinity ligands for selectins, inhibited leukocyte migration by binding to selectins and blocking the signaling pathways of adhesion molecules [89,90]. Following intravenous injection of fucoidans, a significant inhibition of neutrophil release into the abdominal cavity was observed in rats with peritonitis [137]. An imbalance between oxidative reactions and antioxidant defenses has been confirmed as a potential factor in the occurrence and progression of IBD [138]. Additionally, reactive nitrogen species, reactive oxygen species (ROS), and ROS by-products are commonly identified as secondary messengers of apoptosis [139]. Fucoidans, known for their anti-inflammatory properties, reduced colonic levels of myeloperoxidase, NO, and MDA, while increasing the levels of the antioxidant enzymes (SOD and CAT) in a dextran sulfate sodium salt (DSS)-induced acute colitis mouse model [28]. Administration of FCS improved the expression of ZO-1, claudin-1, nuclear factor erythroid 2-related factor 2 (Nrf2), SOD, and NAD(P)H:quinone oxidoreductase 1 (NQO-1) in damaged colon tissue of cyclophosphamide-treated mice, showing significant protective effects against intestinal barrier damage and oxidative stress [140]. The alleviation of IBD symptoms has also been attributed to the restoration of intestinal barrier function and the gut microbiota structure [91,141]. H_2_O_2_-induced disruption of the intestinal epithelial barrier is prevented by fucoidans and FCS through the reduction of disruption of the intestinal epithelial cells, tight junction abnormalities, and increased paracellular permeability [92,93]. Additionally, the colonic fermentation of fucoidans and FCS has been shown to inhibit the colonization of conditional pathogens, lower the pH in the colon lumen, provide an energy source for colonic cells, and reduce the production of ROS [71,140,142].

### 3.7. Anti-Cancer Activity

**Figure 6:**
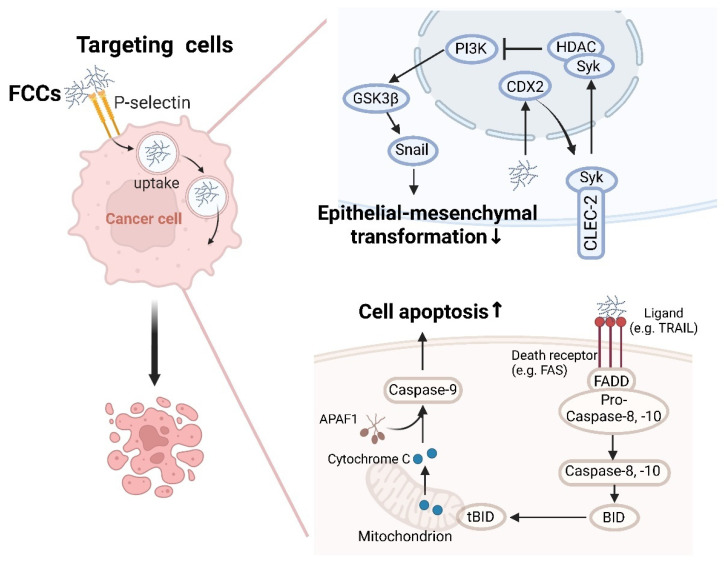
The antitumor mechanisms of FCCs on gastrointestinal cancer. FCCs have been identified as potential anticancer agents against gastric and colon cancers, involving mechanisms of targeting cancer cells, regulating apoptosis and autophagy, and inhibiting tumor metastasis. GSK3β, glycogen synthase kinase 3; PI3K, phosphoinositide 3-kinase; CDX2, caudal-related homeobox transcription factor 2; HDAC, histone deacetylase; Syk, spleen tyrosine kinase; CLEC-2, C-type lectin-like receptor-2; APAF1, apoptotic protease activating factor-1; FADD, fas-associating protein with a novel death domain; TRAIL, TNF-related apoptosis-inducing ligand.

Fucoidans, FCS, and their derivatives have been demonstrated to be potent anticancer agents against tumors of different histogenesis, such as lung, breast, liver, colon, prostate, and bladder cancers [143,144]. The reported anticancer activities of FCCs against gastric and colon cancers primarily involve three mechanisms: targeting cancer cells, regulating apoptosis and autophagy, and inhibiting tumor metastasis (Figure 6).

The activation of caspases is identified as a key mechanism of apoptosis induced by fucoidan. Fucoidan inhibited the growth of human colon cancer cells (HT-29 and HCT-15) and induces apoptosis mediated by caspase activation [38,94]. In both death receptor-mediated and mitochondrial-mediated apoptotic pathways, fucoidan facilitated the binding of death receptor ligands (e.g., TRAIL) to specific death receptors (e.g., Fas and DR5) located on the plasma membrane, resulting in the activation of caspase-8 [145]. This activated caspase-8 subsequently triggers the activation of downstream caspase-3 and/or cleaves Bid, a BH3-only pro-apoptotic member of the Bcl-2 family. The cleaved t-Bid translocates to the mitochondria, enhancing mitochondrial membrane permeability and prompting the release of cytochrome c [146]. The cytosolic cytochrome c associates with apoptotic protease-activating factor 1 (Apaf-1) and inactive procaspase-9 to form the apoptosome, leading to the activation of caspase-9. Kim et al. further demonstrated that the inhibition of caspase-8 and -9 induced a reduction of fucoidan-mediated apoptosis [94]. The low Mw FCS (LFCS) extracted and isolated from sea cucumbers significantly inhibited the growth and metastasis of Lewis lung carcinoma (LLC) cells by activating caspase-3 activity in LLC cells to induce cell cycle arrest [147].

Fucoidan has been reported as a potential anti-cancer agent for preventing the invasive metastasis of cancer cells [95,148,149]. Research has shown that C-type lectin transmembrane receptor 2 (CLEC-2) is highly expressed in normal gastric mucosa, and its loss facilitates epithelial–mesenchymal transformation (EMT) and metastasis of gastric cancer [150]. Data from Xu et al. (2020) suggested that fucoidan targets CLEC-2 to exert anti-tumorigenic and anti-metastatic effects [95]. Fucoidan significantly increased the expression of CLEC-2 in gastric cancer cells by modulating CDX2, a critical regulator of gut homeostasis, thereby inhibiting gastric cancer progression. Moreover, the inhibition of growth, migration, and invasion of various gastric cancer cells by fucoidan can be reversed through CLEC-2 knockdown. CLEC-2 prevents the activation of Ser and Thr kinase (AKT) and glycogen synthase kinase 3β (GSK3β) signaling in a spleen tyrosine kinase (SYK)-dependent manner, as well as the invasiveness and expression of EMT markers in cancer cell lines, which suppressed gastric cancer cell metastasis [151]. FCCs, characterized by the presence of sulfate groups and fucose residues, promote targeted anti-tumor effects or serve as delivery vehicles for other anti-cancer drugs by binding to tumor-specific surface receptors. Heller et al. (2023) reported the use of fucoidan nanoparticles for the delivery of the anti-cancer drug vismodegib [96]. P-selectin is markedly overexpressed in cancers (gastric cancer, colorectal cancer, etc.) and serves as a diagnostic marker [125,126]. Fucoidan is known as a P-selectin targeting agent [90]. These nanoparticles, binding to P-selectin receptors via fucoidan, initiated a transcytosis process that transported drug-loaded nanoparticles from the lumen to the abluminal side of tumor endothelial cells, thereby enhancing the delivery of vismodegib to medulloblastoma tumors. Similarly, DuRoss et al. (2021) demonstrated that fucoidan surface functionalization significantly enhanced the accumulation of a nanoscale metal-organic framework in vitro in CT26.wt murine colon cancer cell lines and in vivo in a CT26.wt tumor-bearing mouse model [97].

## 4. Relationship of Structure and Functional Activity of FCCs

The bioactivity of carbohydrates is influenced by their monosaccharide composition, molecular weight, substituent groups, and glycosidic bond types. This comprehensive study analyzed the impact of molecular weight, sulfate groups, monosaccharide composition, and glycosidic bond types of marine fucoidan on gastrointestinal health, contributing to the targeted development and application of FCCs.

### 4.1. Molecular Weight

Molecular weight is a key factor affecting the activity of marine FCCs. Low molecular weight carbohydrates have been demonstrated to possess superior prebiotic activity, as they are more readily degraded and utilized by gut microbial strains. This is exemplified by the low molecular weight of typical prebiotics such as HMOs, galacto-oligosaccharides (GOS), fructo-oligosaccharides (FOS), and mannan oligosaccharides (MOS) [152,153]. The molecular weight of a carbohydrate influences the selectivity of fermentation. During in vitro fermentation, fucoidan that underwent acidolysis had a positive effect on increasing microbiota density, lowering the pH of the fermentation broth, and producing higher SCFAs compared to the original fucoidan [71].

Both low- and high-Mw fucoidans have been reported to exhibit anti-adhesive properties. The polyanionic and viscous nature of high molecular weight fucoidan products is advantageous in forming anti-adhesion barriers. Chen et al. (2022) reported that fucoidan, with a molecular weight of 400 kDa, can inhibit *H. pylori* adhesion to host cells [75]. Furthermore, both post- and pre-treatment with fucoidan (400 kDa) significantly reduced the *H. pylori* count in the stomachs of mice. Low molecular weight products are considered receptor mimetics or can be further conjugated with macromolecules such as proteins or lipids to form glycomimetic ligands, enhancing their anti-adhesive activity. A prominent example is fucosylated HMOs, known for their anti-adhesive properties, with a degree of polymerization ranging from 3 to 10 [154]. Fucoidan oligosaccharides with molecular weights of 1–3 kDa have been used to conjugate bovine serum albumin as a novel fucosylated glycan that specifically binds to the intestinal pathogen *C. jejuni* [78]. Considering the distinct characteristics of various gastrointestinal pathogens, their classification based on molecular weight serves as a preliminary screening method to specifically obtain electronegative and/or receptor-mimetic dominant anti-adhesives from FCCs.

Azuma et al. (2012) reported the effects of molecular weight on anti-cancer activity, indicating that intermediate Mw fucoidan (IMWF, 110–138 kDa) significantly inhibited tumor growth and increased the survival time of colon 26 tumor-bearing mice compared to low Mw fucoidan (LMWF, 6.5–40 kDa) and high Mw fucoidan (HMWF, 300–330 kDa) [155]. In addition, fucoidan with a molecular weight of 95 kDa has been reported in several studies to significantly inhibit the growth of gastric and colon cancer cell lines, indicating that its superior anti-cancer activity is contributed to by specific Mw fractions such as 95 kDa and 110–138 kDa [38,94,95].

Low, intermediate, and high Mw fractions of FCCs demonstrate advantages in prebiotic effects, anti-cancer activity, and forming protective barriers against pathogens individually. This functional classification based on Mw suggests that tailoring the molecular weight of FCCs could optimize their health benefits to develop targeted intervention products for specific health issues.

### 4.2. Sulfate Groups

Marine-derived FCCs are characterized by a high sulfate group content, which promotes the selectivity of FCCs fermentation. Species containing exo- and endo-sulfatases are capable of desulfating carbohydrates [156]. Free sulfates may be cross-fed by sulfate-reducing bacteria, such as *Desulfovibrio* and species of the *Proteobacteria* phylum, forming hydrogen sulfide (H_2_S) [157]. High concentrations of H_2_S inhibit mitochondrial complex IV, reducing butyrate oxidation and oxygen consumption, which disrupts the local anaerobic environment and favors the overgrowth of facultative anaerobic bacteria [158]. Therefore, in studies utilizing highly sulfated FCCs as prebiotics, the dosage of carbohydrates must be carefully considered. Different sulfation patterns influence the outcomes of carbohydrate colonic fermentation. It has been reported that Dfuc-Ib, a fucoidan predominantly containing 2-O-sulfo groups, increased the abundance of *Proteobacteria* in HFD-fed models, while Dfuc-Pg, predominantly containing 4-O-sulfo groups, maintained a well-balanced gut microbiota profile [159]. The presence of sulfate groups is crucial for anti-pathogen adhesion activity. Forming an anti-adhesive barrier through the interaction of the negatively charged polymer with pathogen surfaces or positively charged host cell receptors is one of the anti-adhesive mechanisms of sulfated polysaccharides/oligosaccharides. Fucoidan from *Cladosiphon* and *Fucus* and dextran sulfate sodium inhibited *H. pylori* attachment to porcine gastric mucin at pH 2.0, while non-sulfated polysaccharides, such as mannan and dextran, did not exhibit adhesion inhibition [76].

The degree of sulfation did not play a role in the inhibition of colony formation in cancer but may be involved in inhibiting cancer cell metastasis. Galactofucan SmF3 (with 35% sulfate content) demonstrated colony formation inhibition in colon cancer DLD-1 cells, and no change in this inhibitory ability was observed after desulfation [20]. The interaction between cancer cells and extracellular matrix (ECM) proteins is a fundamental step in the progression of metastasis. Fucoidan inhibits cancer cell adhesion to fibronectin, an ECM protein, and this effect is lost when fucoidan is desulfated [160]. Sulfate substitutions in FCCs play a key role in inhibiting cancer cell metastasis and the formation of pathogen barriers, while a high sulfate content may lead to the excessive production of sulfate metabolic byproducts, such as H_2_S, during colonic fermentation. Balancing the dual effects of FCCs requires careful consideration of both dosage and administration routes.

### 4.3. Monosaccharide Composition

The monosaccharide composition also affects the microbial community structure in the fermentation of FCCs. The monosaccharide components of FCS include GlcA, GalNAc, and fucose, with the proportion of each monosaccharide varying across different FCS samples [14,56]. Sulfated fucans and fucose-rich EPS usually contain a high proportion of fucose residues, as well as varying proportions of other neutral and acidic monosaccharides, including galactose, mannose, glucose, xylose, GlcA, and galacturonic acid [161,162]. In studies on in vitro fermentation by human gut microbiota, the proportion of fucose content influences the regulatory effect of FCS on the microbial community structure [8,163]. FCS-*pg*, composed of GlcA, GalNAc, and fucose in a ratio of 1.0:0.8:1.5, was mainly fermented by *Bacteroides*. fCS-*Sc* (GlcA/GalNAc/fucose = 0.90/1.00/1.08) increased the abundance of *Megamonas*, *Bacteroides*, *Fusobacterium*, *Parabacteroides*, *Prevotella*, and *Faecalibacterium*. Two fucoidans (FuA and FuL) are composed of similar fucoses (58.6 mol% and 54.1 mol%), have similar sulfate contents (21 mol% and 18.4 mol%), and different proportions of monosaccharide composition [164]. FuA with a high GlcA content (24.1%) stimulated the growth of Ruminococcaceae in Wistar rats, while FuL with a high galactose content (19.3%) increased the abundance of *Lactobacillus*, *Anaeroplasma*, and *Thalassospira*.

A complex monosaccharide composition enhances the ability of sulfated fucans to act as host cell receptor analogs, serving as decoy receptors for pathogens. The structure of the fucosylated Lewis b (Le^b^) antigen, recognized by *H. pylori* adhesins BabA and AlpA, includes fucose, galactose, *N*-acetylglucosamine, and glucose [100]. Fucoidan, which inhibited *H. pylori* adhesion to host cells, had a monosaccharide composition closely matching that of the Le^b^ antigen in experiments with AGS cells and BALB/c mouse models [75]. Its monosaccharide components contained 51.2 mol% fucose, 16.2 mol% hexuronic acid, 15.8 mol% galactose, 12.2 mol% glucose, and 4.6 mol% GalNAc. It has been reported that fucoidan with a high fucose content inhibits leukocyte migration to inflammation sites by binding to selectins and blocking the signaling of adhesion molecules [89,90]. Semenov et al. (2020) extracted and isolated two types of fucoidan, Fv_crude and FE_F3, with differing fucose contents (25 mol% and 87 mol%, respectively), from *Fucus distichus* subsp. *evanescens* [165]. Interestingly, both extracts (Fv_crude and FE_F3) reduced monocyte adhesion to the endothelial cell layer and lowered the gene levels of tested MNC-associated markers, but the effects were most pronounced when cells were treated with FE_F3 [166].

FCCs characterized by a high fucose content show a specific response to fucose-utilizing species and cell surface receptors in the body, exhibiting microbiota regulatory, anti-pathogenic adhesion, and anti-inflammatory activities. The presence of other monosaccharides, including galactose and mannose, promotes the diversity and richness of host microbiota and the potential of FCCs to target diverse biological sites in vivo. The contributions of complex monosaccharide compositions to other activities, such as anti-cancer effects, require more comprehensive and in-depth research into the structure–activity relationships of FCCs.

### 4.4. Type of Glycosidic Bond

As mentioned in Section 3.2, the backbone of l-fucopyranose residues and complex branching in sulfated fucans provided a growth and colonization advantage for specific species, especially *Bacteroides* spp. with CAZyme-encoding genes for hundreds of different GHs and PLs [25]. Sugar chains with different glycosidic linkages are widely known to be hydrolyzed by distinct enzymes, which further affects the fermentation selectivity of FCCs. Terminal α-l-fucose residues from the fucoidan backbone can be cleaved by α-l-fucosidases present in *Bacteroides* spp., which are primarily found in glycoside hydrolase (GH) families 29, 95, and 141 [167]. In the CAZyme database, α-l-fucosidase (EC 3.2.1.51) in GH 29 and GH 141 families is used by *Bacteroides* to cleave α-l-fucoside from fucoidan and convert it into carbon sources (fucose and alcohol). Additionally, α-1,3-l-fucosidase (EC 3.2.1.111), which hydrolyzes (1→3)-linkages between α-l-fucose and *N*-acetylglucosamine residues, assists *Bacteroides* in degrading fucoidan with *N*-acetylglucosamine residue branches. α-1,2-l-fucosidase (EC 3.2.1.63) from the GH95 family of *Bacteroides* exhibits specificity for α-1,2-linked l-fucopyranose in carbohydrates, such as FCS. The glycosidic bond is not a decisive factor for the efficacy of fucosyl-polysaccharides according to the current study. Fucoidan containing backbones of α(1→3)-linked and/or α(1→4)-linked α-l-fucopyranose both inhibited colony formation of colon cancer cells (DLD-1) [98]. In addition, the study by Thinh et al. (2013) showed that α(1→3) linkage mode fucoidan with a side chain of 1,4-linked 3-sulfated α-l-Fuc*p* and 6-linked galactose also yielded anticancer activity against DLD-1 cells [20]. The types of glycosidic bonds in many marine FCCs that promote gastrointestinal health are not sufficiently clear for further in-depth analysis, particularly regarding branched glycosidic bonds and the orientation of the hydroxyl group. Most research has focused on the relationship between the primary structure and functional activity, while discussions on the composition of glycosidic bonds in sugar chains and their complex spatial structures remain limited. Further investigations of FCCs are required to elucidate their fine structure and explore the relationships between various structural factors and their regulation of gastrointestinal health.

## 5. The Potential Limitations Associated with FCCs

FCCs, characterized by their complex carbohydrate structure and high fucose content, pose several challenges for future research and applications. These challenges include bioavailability, potential side effects, difficulties in large-scale production, and the need for standardized FCC formulations.

Current evidence suggests that the overall recovery rate of GFS (300 kDa) in the digesta and sediment fraction of the intestinal chyme post in vitro digestion is 89.2–93.8%, suggesting a potential bioavailability of less than 10% through the oral, gastric, and intestinal phases [168,169]. In a transcellular transport model of intestinal epithelial cells, the absorption rate of fucoidan (Sigma) was less than 30%, primarily relying on clathrin-mediated endocytosis [170]. Fluorescently labeled FCS showed no absorption in intestinal epithelial cell transport models, indicating that epithelial cells may struggle to transport and absorb FCS [93]. Low Mw fucoidan (2.5 to 10 kDa) has been shown to enhance bioavailability in vivo, with a 2-fold increase in fucose content in rats compared to medium Mw fucoidan (>10 kDa) [171]. Future research could consider appropriately reducing the molecular weight of FCCs to achieve higher bioavailability and better therapeutic efficacy. While the restricted gastrointestinal transport and absorption of fucoidan enhance its probiotic and anti-pathogenic adhesion properties, its low bioavailability necessitates high doses for effective tissue inflammation and cancer treatment, increasing the risk of potential side effects. Chun et al. demonstrated that administering high doses of fucoidan (2000 mg/kg) significantly increased thyroid weight in rats, indicating potential hepatotoxicity [172]. Additionally, a dosage of 6 g/day of fucoidan caused diarrhea in some patients in a clinical study, which subsided after discontinuation [173]. The doses of FCCs reported in current studies are significantly below the levels associated with potential side effects, and most studies indicate that FCCs exhibit low biological toxicity [30,42,72]. An in vivo experiment using SD rats tested the toxicity of oral fucoidan [174]. Rats were gavaged with 150–1350 mg/kg fucoidan daily for 28 days. The experimental results showed no apparent abnormalities in the vital signs of rats, except for a slight increase in serum urea nitrogen in females.

Standardizing fucoidan-containing formulations remains a significant challenge for their entry into high-value markets. Marine sources of fucoidan, such as brown algae and sea cucumbers, exhibit seasonal and geographical variability in structural composition and yield [175]. Environmental factors, including sampling season, nutrient availability, salinity, blade age, and reproductive stage, influence the carbohydrate composition of seaweed tissues [175,176]. Moreover, fucoidan is not the primary component in seaweed/sea cucumber tissues, and downstream processing is complicated by the presence of pigments, proteins, lipids, polyphenolic compounds, alginates, starch, and other interfering substances [176]. The inherent heterogeneity of FCCs and the presence of co-extracted interfering substances hinder the standardization of formulations, impacting the reproducibility of preparation processes and the reliability of subsequent bioactivity studies. Heterologous expression of enzymes involved in fucoidan synthesis and degradation offers a promising approach to producing products with controlled structural characteristics. For example, Chen et al. successfully expressed endo-1,4-fucanase (FunA) in *Escherichia coli*, yielding a specific tetrasaccharide product (→3-α-l-Fuc*p*-1→3-α-l-Fuc*p*2,4(OSO_3_^−^)-1→3-α-l-Fuc*p*2(OSO_3_^−^)-1→3-α-l-Fuc*p*2(OSO_3_^−^)-1→) [177]. FunA is an endo-1,4-fucanase that cleaves α-1,3 glycosidic bonds between 2-O-sulfated and non-sulfated fucose residues and exhibits transglycosylation activity with glycerol, methanol, and l-fucose as acceptors [178].

Large-scale production of FCCs should prioritize appropriate raw material sources, stable extraction/degradation methods, and stringent purity requirements. Commercial fucoidan is predominantly derived from two Atlantic brown algae species: *Fucus vesiculosus* and *Ascophyllum nodosum*. However, FCS and fucose-rich EPS currently lack low-cost and standardized production materials [179]. The discovery of novel species and the development of artificially cultured cell lines are crucial for expanding the commercial availability of high-value FCCs [41,180]. Various downstream processing techniques, including pretreatment, extraction, purification, degradation, and enzymatic modification, significantly influence both the biological activity research and industrial applications of FCCs [181]. Stable extraction and degradation methods are essential for industrial-scale production and minimizing product structure variations. Non-mild processes, such as acid treatment and organic solvent treatment, can directly affect the fucose content and sulfate group retention rate in FCCs, necessitating careful selection and optimization of process conditions [71,107]. FCC products often contain impurities, such as proteins and polyphenols, which can hinder accurate evaluation of their biological activity [182,183]. Novel purification techniques, including molecularly imprinted polymer (MIP)-based selective solid-phase extraction and metachromasia-affinity-based chromatography, offer high selectivity, environmental friendliness, and speed, making them promising alternatives to traditional ion-exchange chromatography and conventional purification processes [184,185,186].

This review summarizes both in vivo and in vitro studies demonstrating the various gastrointestinal activities of FCCs. Large-scale cohort studies have been conducted primarily in the context of *H. pylori* eradication therapy. Given the high prevalence of *H. pylori* in China (estimated at 44.2%, affecting approximately 589 million individuals), there is a substantial volunteer base for clinical studies in this area [187]. A real-world clinical study involving 122 participants demonstrated the efficacy of a fucoidan-containing drink in clearing *H. pylori* [77]. Additionally, an open-label randomized controlled trial with 86 participants revealed significant alleviation of gut microbiota dysbiosis with fucoidan supplementation during *H. pylori* eradication therapy [73]. While clinical data support the efficacy of FCCs in other conditions, such as gastric ulcers, the number of patients included in these studies is significantly smaller than those in *H. pylori* eradication trials [87]. Most of the activities attributed to FCCs in this review, including effects on gut microbiota composition and metabolic regulation, anti-pathogenic adhesion, and anti-cancer activities, have been explored through in vitro fermentation, cell, and animal models. Further clinical validation is needed. 

The doses of FCCs used in various studies vary, influenced by factors such as model selection, the structural characteristics of FCCs, and the method of administration (Table 1). The diversity in FCC structures, model selection, and administration methods across studies may introduce potential biases into the experimental outcomes. Variations in FCC doses are attributed to differences in models and experimental designs. For example, Shibata et al. (2003) administered 5% fucoidan in the drinking water of gerbils inoculated with *H. pylori*, while Chen et al. (2022) administered 800 mg/kg/day of fucoidan via oral gavage to mice prior to infection with *H. pylori* [75,76]. Indeed, these differences in experimental design should be carefully considered when interpreting and applying the findings of these studies.

This section addressed the challenges associated with the structure, processing, and research methodology when considering the biological activities and industrial applications of FCCs. The trade-off between bioavailability and safety emphasizes balancing therapeutic efficacy with minimal adverse effects. The variability arising from environmental and processing factors presents challenges for standardizing FCC formulations and large-scale production, highlighting the potential benefits of biotechnological approaches. Furthermore, conducting large-scale cohort studies would provide more substantial evidence for the gastrointestinal health benefits and broader application prospects of FCCs.

## 6. Conclusions and Prospects

Algae, invertebrates (sea cucumber and sea urchins), microalgae, and bacteria within marine habitats constitute abundant sources of FCCs. Marine-derived FCCs, in particular sulfated fucoidan, FCS, and fucose-rich EPS, have attracted significant attention due to their abundant sources, good biocompatibility, minimal toxic side effects, high stability, and diverse activities. Marine-derived FCCs have been revealed to have significant potential in promoting gastrointestinal health, including shaping gut microbial communities, inhibiting adhesion of *H. pylori* and gut pathogens, regulating microbial metabolism, and ameliorating gastrointestinal inflammatory injuries and cancer.

The common structural characteristics of FCCs from different marine species include a high molar ratio of fucose in a complex monosaccharide composition and various modifications of substitutions, especially sulfate groups. Serving as decoy receptors, the terminal fucose residues of FCCs competitively bind to *H. pylori* adhesins, pathogen adhesion factors, and signaling molecules involved in leukocyte migration to inflammation sites, demonstrating a distinct advantage in anti-adhesion and anti-inflammation activities. Additionally, the polyanionic properties attributed to the presence of sulfate groups and uronic acids further enhance the anti-adhesive properties of FCCs. The glycosidic bonds between fucose residues in FCCs provide a growth and colonization advantage for *Bacteroides*, improving gut microbiota dysbiosis induced by metabolic disorders and antibiotic therapy. For FCCs, large molecular polymers contribute to the formation of anti-adhesion barriers, while the polysaccharide degradation products are able to be efficiently utilized by gut microbiota, suggesting that molecular weight fractionation provides a feasible approach for the targeted screening of FCCs. While the contributions of individual structural features to the bioactivity of FCCs have been reported, the intricate structural details of FCCs, such as glycosidic linkages, sulfation patterns, and complex monosaccharide composition, are often overlooked. Despite the challenges of detailed structural analysis and the difficulties in investigating structure–activity relationships, the complex structural details enhance the diversity of physiological activities of FCCs. One suggested direction for future work is to consider incorporating complex rather than individual structural characteristics into structure–activity relationship research, which would be crucial for unlocking the full potential of these compounds in promoting gastrointestinal health and treating diseases.

The complex sources, heterogeneous structural compositions, unstable downstream processes, insufficient clinical evidence, and potential biases in research methodologies present numerous challenges for the industrial application of FCCs. Emerging biotechnologies, such as artificially cultured cell lines and affinity chromatography purification, are continuously being explored to address the limitations associated with FCCs. In summary, marine-derived FCCs, characterized by diverse and complex structures, have significant potential in improving gastrointestinal health and are expected to serve as sustainable nutritional or functional foods for the prevention and treatment of gastrointestinal disease.

## Figures and Tables

**Table 1:** Structural information and impact on the gastrointestinal health of fucose-containing carbohydrates from different sources.

No.	FCCs	Source	Molecular Weight (kDa)	Monosaccharide Composition	Fucose Content (%)	Sulfate Group Content (%)	Structure	Model (Dose)	Effect	Reference
1	Fucoidan	*Laminaria japonica*	654.55	Fuc/Gal/Rha/GlcA/GalA/Glc/Xyl/Man = 55.55/16.32/7.09/5.14/3.35/3.64/4.71/4.19	55.55 ± 1.19	33.68 ± 0.46	——	In vitro (10 mg/mL)	Improve the abundance of Bacteroides	[71]
2	HMAF	*Laminaria japonica*	1.5–20	Fuc/Gal/Rha/GlcA/GalA/Glc/Xyl = 14.62/18.57/0.73/42.49/10.58/6.03/6.98	14.62 ± 0.13	26.03 ± 0.79	——	In vitro (10 mg/mL)	Improve the abundance of *Bacteroides*, *Bifidobacterium*, *Lactobacillus*, and *Megamonas*; improve the production of SCFAs (acetate and propionate)	
3	LMAF	*Laminaria japonica*	<1.5	Fuc/Gal/Rha/GlcA/GalA/Glc/Xyl/Man = 62.83/14.67/3.17/5.56/0.37/3.80/4.67/4.93	62.83 ± 0.86	30.51 ± 0.45	——	In vitro (10 mg/mL)	Promote level of butyrate, indole-3-propionic acid, and indole-3-carboxaldehyde	
4	fCS-Sc	*Stichopus chloronotus*	111	GlcA/GalNAc/Fuc = 0.90/1.00/1.08	36.24	51.6	——	In vitro (10 mg/mL)	Increase the beneficial bacteria; suppress pathogenic microorganisms; promote SCFA production	[8]
5	FuL	*Laminaria japonica*	310	Man/GlcA/Glc/Gal/Xyl/Fuc = 11.2/7.3/5.2/19.3/2.9/54.1	54.1	18.4	Linkage mode: α(1→3)	HFD-induced obese mice (200 mg/kg)	Improve diet-induced metabolic syndrome (MetS); increase *Bacteroides* and *Akkermansia*	[42]
6	FuA	*Ascophyllum nodosum*	1330	Man/GlcA/Glc/Gal/Xyl/Fuc = 7.3/24.1/1.5/7.2/1.3/58.6	58.6	21	Linkage mode: α(1→3)/α(1→4)	HFD-induced obese mice (200 mg/kg)	Increase the amount of *Akkermansia* during alleviation of MetS	
7	FUC	*Undaria pinnatifida*	309	——	20.3	27.8	α-1,3-linked-l-fucose-4-sulfate	HFD-induced obese mice (100 mg/kg)	Increase the BSH activity in the ileal content	[72]
8	SFP	*Sargassum fusiforme*	703	Fuc/Gal = 73.16/26.84	73.16	23.6	1,3-, 1,4-, 1,3,4-linked-α-l-Fuc*p* and 1,3-, 1,6-linked-β-d-Gal*p* with partial sulfation at C-4, C-3 of fucose units and C-6, C-3 of galactose units	HFD-induced obese mice (100 mg/kg/d)	Beneficial effects on disorders of intestinal microbiota (decreasing the ratio of Firmicutes/Bacteroidetes (F/B))	[19]
9	fCS-Ib	*Isostichopus badionotus*	10.9	GlcA/GalNAc/Fuc = 1.43/1/1.71	41.3	——	——	High-fat and high-fructose diet-induced obese mice (20 mg/kg/d)	Reverse the increase of F/B value induced by obesity	[56]
10	Am-CHS	*Acaudina molpadioides*	21.53	GlcA/GalNAc/Fuc = 1/1.14/1.55	42	27.81	——	HFD-induced obese mice (80 mg/kg)	Decrease in Bacteroidetes, increase in Firmicutes, and elevation in SCFA-producing bacteria	[30]
11	Fucoidan	*Undaria pinnatifida*	220	——	23.05 ± 0.2	26.84 ± 0.3	——	Standard quadruple therapy (SQT) for patients (positive for *H. pylori*) (2000 mg/d)	Improve gut dysbiosis during SQT for *H. pylori* eradication	[73]
12	HLP	*Holothuria leucospilota*	52.8	Rha/Fuc/GlcA/Gal/Glc/Xyl = 39.08/35.72/10.72/8.43/4.23/1.83	35.72	——	——	Type 2 diabetes mellitus rats (100–200 mg/kg)	Increase the short-chain fatty acid-producing bacteria and decrease the opportunistic bacterial pathogen	[74]
13	Fu	*Sargassum hemiphyllum*	440	Fuc/hexA/Gal/Glc/GalNAc = 51.2/16.2/15.8/12.2/4.6	51.2	12.87	——	AGS cell (250–2000 μg/mL); BALB/c mice (800 mg/kg/d)	Inhibit adhesion of *H. pylori* (Hp) and total count in vitro; reduce the count of Hp in vivo	[75]
14	Fucoidan	*Cladosiphon okamuranus*	——	——	——	——	——	In vitro (2–2000 μg/mL); Mongolian gerbils (0.05 and 0.5%)	Inhibit the Hp attachment in vitro; reduction of *H. pylori*-induced gastritis and the prevalence of Hp infected animals	[76]
15	FPD	*——*	——	——	——	——	——	Patients with confirmed Hp infection (2 mg/d)	Eradication and clearance of Hp at the rate of 77.6% (66/85) and 20.0% (17/85), respectively, after 4 weeks treatment	[77]
16	Ofuc	*Macrocystis pyrifera*	1–3	——	——	——	——	In vitro (20 μg/mL)	Recognition by specific *Ulex europaeus* fucose-specific lectin and *Campylobacter jejuni*	[78]
17	fucoidan	*Fucus vesiculosus*	<200	Fuc/Gal/Xyl/Man = 89.6/5.2/3.9/1.4	89.6	26	——	In vitro (2.40 and 24.00 mg/mL)	Inhibit the binding of hNoV GII.4 virus-like particle (VLPs) to type A saliva	[79]
18								Zebrafish (*Danio rerio*) larvae (6.00 mg/mL)	Inhibit the replication of human noroviruses (hNoV) GII.4[P16] in zebrafish	[80]
19	Galactofucan	*Sargassum fusiforme*	21.33	Man/Rha/GlcUA/Gal/Fu = 0.31/0.40/0.53/0.82/1.00	32.7	16.08	——	In vitro (3–12 mg/mL)	Prevent the binding of NoV GII.4 virus-like particles to histo-blood group antigens	[81]
20	FeF	*Fucus evanescens*	160	Fuc/Gal = 9/1	90	28	→3)-α-l-Fuc*p*(2,4OSO_3_^−^)-(1→4)-α-l-Fuc*p*(2OSO_3_^−^)-(1→) and (→3)-α-l-Fuc*p*(2OSO_3_^−^)-(1→4)-α-l-Fuc*p*(2OSO_3_^−^)-(1→	African green monkey kidney (Vero) cells (0.2–2000 μg/mL)	Moderate replication inhibition against ECHO-1 (enterovirus)	[82]
	FeHMP		50.8	Fuc/Gal = 1/0	100	40	→3)-α-l-Fuc*p*(2,4OSO_3_^−^)-(1→4)-α-l-Fuc*p*(2,4OSO_3_^−^)-(1→			
21	Fv	*Fucus vesiculosus*	20–200	——	33	23	——	In vitro (100 mg/mL)	Antimicrobial activity against *Candida albicans*	[83]
22	Fucoidan (Sigma)	*Fucus vesiculosus*	95	——	——	——	——	Ulcer rat model induced by aspirin (20 mg/kg)	Protect against oxidative damage to gastric mucosa during ulceration	[84]
23	Fucoidan (Sigma)	*Fucus vesiculosus*	——	——	——	——	——	Ulcer rat model induced by ethanol (25 and 50 mg/kg)	Mitigation of gastric ulceration severity	[85]
24	FucoMax^®^	*Undaria pinnatifida; Laminaria saccharina; Cladosiphon okamuranus*	200	——	——	——	Linkage mode: α(1→2)/α(1→4)	Ulcer rat model induced by ethanol (50 mg/kg)	Protection against ethanol-induced gastric mucosal damage in rats	[86]
25	Fucoidan	*Cladosiphon Okamuranus*	——	——	——	——	——	Patients with gastric ulcers (100 mg/d)	Reduce ulcer symptoms and protection against the oxidative damage to gastric mucosa during ulceration	[87]
26	Fucoidan	*Scytosiphon lomentaria*	∼103 & ∼460	Man/Gal/Fuc/galA/glu/Xyl/Rha/GlcA/17.1/13.6/15.9/4.7/3.3/5.7/1.0/1.1	25.4	15.5	——	DSS and FD-fed ulcerative colitis mouse model (100 mg/kg)	Improve UC symptoms, oxidative stress, colonic inflammation, and tight junction dysfunction	[88]
27	Fucoidan (Sigma)	*Fucus vesiculosus*	——	——	——	——	——	T84 cells (50 μg/mL)	Inhibit cell adhesion to CD11b/CD18 and leukocyte migration	[89]
28	Fucoidan	*Laminaria saccharina*	——	——	40.5	36.7	——	Rats with peritonitis (0.7 μg/d)	Inhibit neutrophil release into the abdominal cavity	[90]
29	MPF	*Macrocystis pyrifera*	66	Fuc/Xyl/Man/Gal/Glu/Ara/Rha = 66.2/3.1/5.9/15.6/3.9/2.3/3.0	66.2	25.7	——	DSS-induced acute colitis mouse model (400 mg/kg)	Reduce oxidative stress and inflammation in DSS-induced colitis	[28]
	DP-MPF		17.4	Fuc/Xyl/Man/Gal/Glu/Ara/Rha = 62.9/4.5/8.1/14.3/6.9/0.4/3.0	62.9	19.7	——			
30	Fucoidan	*Fucus vesiculosus*	170	——	38	20	——	DSS-induced acute colitis mouse model (100 and 300 mg/kg)	Enhance disease recovery; bolster intestinal barrier function	[91]
31	Fucoidan	*Cladosiphon okamuranus*	——	——	——	——	——	Caco-2 cells (2.5 mg/mL)	Prevent H_2_O_2_-induced disruption of the intestinal epithelial barrier	[92]
32	fCS-Sc	*Stichopus chloronotus*	111	GlcA/GalNAc/Fuc = 0.90/1.00/1.08	36.24	51.6	——	Caco-2 cells (25–400 μg/mL); intestinal oxidative injury mouse model (50–200 mg/kg)	Enhance intestinal barrier function; mitigate oxidative stress in vitro and in vivo	[93]
33	Fucoidan (Sigma)	*Fucus vesiculosus*	——	——	——	——	——	HT-29 cell; HCT116 cell (0–20 μg/mL)	Suppress human colon cancer cell growth and induction of apoptosis	[94]
34	Fucoidan (Sigma)	*Fucus vesiculosus*	95	——	——	——	——	HT29 colon cancer cell (0–150 μg/mL)	Inhibit HT29 colon cancer cell growth	[38]
35	Fucoidan (Sigma)	*Fucus vesiculosus*	——	——	——	——	——	AGS, MGC80-3 and HGC-27 cells (10 mg/mL)	Suppress gastric cancer cell growth, migration, and invasion	[95]
36	Fucoidan (Sigma)	*Fucus vesiculosus*	——	——	——	——	——	Sonic hedgehog medulloblastoma animal model (——)	Enhance vismodegib delivery to medulloblastoma tumors via fucoidan binding to P-selectin	[96]
37	Fucoidan (Sigma)	*Fucus vesiculosus*	——	——	——	——	——	CT26.wt murine (1.85 mg/kg); CT26.wt cells (2.5 mg/mL)	Improve tumor control (volume, density, and growth rate) and survival in a syngeneic CRC	[97]
38	ShF1	*Sargassum hornery*	——	Fuc/Gal/Rha/Xyl = 1/0.1/0.09/0.04	81.3	14.9	(1,3)-linked α-l-fucopyranose residues with sulfate groups at positions 2	DDL human colon cancer cell line (50–400 μg/mL)	Colony formation inhibition in DLD-1 colon cancer cells by ShF1, EcF1, and CcF1, with potencies of 44%, 50%, and 55%, respectively	[98]
39	EcF1	*Eclonia cava*	——	Fuc/Gal/Man/Rha = 1/0.21/0.05/0.16	70.4	19.1	Sulfated 1,3-α-l-fucan			
	CcF	*Costaria costata*	——	Fuc/Gal/Man/Rha/Xyl = 1/0.83/0.01/0.05/0.06	51.2	18.9	Sulfated and acetylated galactofucan except for C6 of galactose residues			
	SmF3	*Sargassum mcclurei*	——	Fuc/Gal = 58.5/41.5	58.5	35	→3)-α-l-Fuc*p*(2,4SO_3_^−^)-(1→3)-α-l-Fuc*p*(2,4SO_3_^−^)-(1→ motif with 1,4-linked 3-sulfated α-l-Fuc*p* inserts and 6-linked galactose on reducing end	DDL human colon cancer cell line (50–200 μg/mL)	Colony formation inhibition in colon cancer DLD-1 cells	[20]

——, not determined.

## Data Availability

No new data were created or analyzed in this study. Data sharing is not applicable to this article.
